# Down-Regulation of *NDRG1* Promotes Migration of Cancer Cells during Reoxygenation

**DOI:** 10.1371/journal.pone.0024375

**Published:** 2011-08-30

**Authors:** Liang-Chuan Lai, Yi-Yu Su, Kuo-Chih Chen, Mong-Hsun Tsai, Yuh-Pyng Sher, Tzu-Pin Lu, Chien-Yueh Lee, Eric Y. Chuang

**Affiliations:** 1 Graduate Institute of Physiology, National Taiwan University, Taipei, Taiwan; 2 Institute of Biotechnology, National Taiwan University, Taipei, Taiwan; 3 Graduate Institute of Clinical Medical Science, China Medical University, Taichung, Taiwan; 4 Graduate Institute of Biomedical Electronics and Bioinformatics, National Taiwan University, Taipei, Taiwan; National Cancer Institute, United States of America

## Abstract

One characteristic of tumor microenvironment is oxygen fluctuation, which results from hyper-proliferation and abnormal metabolism of tumor cells as well as disorganized neo-vasculature. Reoxygenation of tumors can induce oxidative stress, which leads to DNA damage and genomic instability. Although the cellular responses to hypoxia are well known, little is known about the dynamic response upon reoxygenation. In order to investigate the transcriptional responses of tumor adaptation to reoxygenation, breast cancer MCF-7 cells were cultured under 0.5% oxygen for 24 h followed by 24 h of reoxygenation in normoxia. Cells were harvested at 0, 1, 4, 8, 12, and 24 h during reoxygenation. The transcriptional profile of MCF-7 cells upon reoxygenation was examined using Illumina Human-6 v3 BeadChips. We identified 127 differentially expressed genes, of which 53.1% were up-regulated and 46.9% were down-regulated upon reoxygenation. Pathway analysis revealed that the HIF-1-alpha transcription factor network and validated targets of C-MYC transcriptional activation were significantly enriched in these differentially expressed genes. Among these genes, a subset of interest genes was further validated by quantitative reverse-transcription PCR. In particular, human N-MYC down-regulated gene 1 (*NDRG1*) was highly suppressed upon reoxygenation. NDRG1 is associated with a variety of stress and cell growth-regulatory conditions. To determine whether *NDRG1* plays a role in reoxygenation, NDRG1 protein was overexpressed in MCF-7 cells. Upon reoxygenation, overexpression of *NDRG1* significantly inhibited cell migration. Our results revealed the dynamic nature of gene expression in MCF-7 cells upon reoxygenation and demonstrated that *NDRG1* is involved in tumor adaptation to reoxygenation.

## Introduction

Tumor populations need to overcome distinct microenvironmental barriers prior to metastasizing to other organs. Invasive cancers, therefore, could be viewed as a series of adaptations in phenotype to their microenvironments. All tumor microenvironments are characterized by nutrient deprivation, low pH, and hypoxia [1]. These changes were linked to perfusion deficits in solid tumors, which came from rapid tumor growth and profoundly disorganized vasculature [2]. It has been suggested that the tumor microenvironment is a unique setting for tumor progression, which requires genetic adaptations in cancer cells for further survival and proliferation. Cell stresses induced by the microenvironment, especially hypoxia [3], [4] and reoxygenation [5], [6], might cause these genetic changes.

Regions of hypoxia are a common feature in solid tumors. Oxygen is a limiting factor because of the imbalance between O_2_ delivery and consumption [7]. The O_2_ deficiency is attributed to insufficient vasculatures and oxygen depletion in successive cell layers distal to the vessel lumen; simultaneously, there is an increase in O_2_ consumption due to the high metabolic rate of tumor cells. Many studies have reported that hypoxic tumors were more malignant and resistant to therapy, and thus had a worse prognosis [8]. This phenomenon has been demonstrated in many tumor types [9], [10].

Moreover, the oxygen concentration within a hypoxic region is highly variable. Since tumor vasculatures are highly inefficient and unstable, red blood cells flux to the hypoxic regions, resulting in reperfusion or reoxygenation [11]. Reoxygenation not only increases oxygen supply but also induces oxidative stress in the cells. This oxidative stress could cause damage to cellular macromolecules and lead to increased genomic instability [12]. If tumor cells survive after exposure to hypoxia/reoxygenation insults, they may demonstrate increases in malignancy [13], DNA over-amplification [14], drug resistance [15], and metastatic potential [16].

Cellular adaptation to hypoxia is well documented, but little is known about adaptive mechanisms to reoxygenation. Therefore, we used genome-wide expression microarrays to investigate the dynamics of transcriptional profiling during reoxygenation in MCF-7 breast cancer cells. Our microarray results showed that N-MYC down-regulated gene 1 (*NDRG1*) had the maximal response after reoxygenation. Therefore, we focused on investigating its functional role in reoxygenation. The functional assays revealed that cell migration of breast cancer cells during reoxygenation was driven by down-regulation of *NDRG1*. Lastly, the regulatory model of *NDRG1* using *in silico* analysis was proposed for further investigation.

## Results

### Identification of genes responsive to reoxygenation

MCF-7 human breast cancer cells were incubated under hypoxia (0.5% O_2_ concentration) for 24 h and then shifted to normoxia. Cells were harvested respectively at 0 (hypoxia control), 1, 4, 8, 12 and 24 h after reoxygenation. Each time series was independently carried out in triplicate. After extracting total RNA, Illumina Human-6 v3 BeadChips were used to examine the dynamics of transcriptional profiling upon reoxygenation. Background-adjusted signals were normalized by a quantile normalization algorithm. In order to identify differentially expressed genes, Student's t-test was used to examine the expression levels of every time point after reoxygenation versus that of time zero. The genes responsive to reoxygenation were selected by choosing genes whose mean *P*-value at a given time point was <10^−4^. In total, we identified 127 genes whose transcript levels deviated significantly from time zero. Among them, 53.1% were up-regulated and 46.9% were down-regulated upon reoxygenation. Most of these genes (n = 112) were identified at only one time point, but 13 were identified at two time points, and two genes were identified at more than two time points.

**Figure 1:**
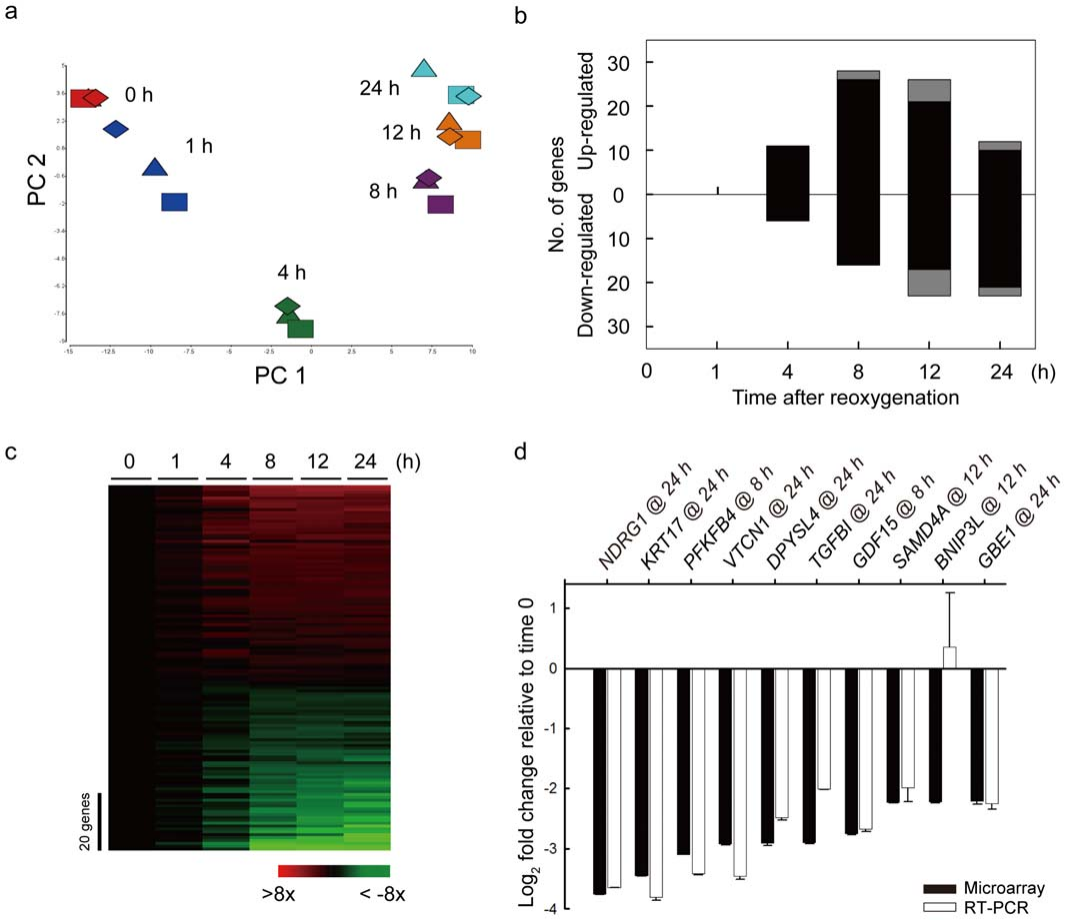
Dynamics of differentially expressed genes during acclimatization to reoxygenation. (a) Principal component analysis (PCA) of O_2_-responsive genes in MCF7 cells during 24 h of reoxygenation after hypoxia. The axes are the first two principal components (PC), which can explain most of the gene expression profiling. Three independent experiments were done at each time point. Different shapes represent different replicates; different colors represent different time points. (b) Number of O_2_-responsive genes at each time point during reoxygenation. Both up-regulated and down-regulated genes are plotted as a function of time. Black bars indicate the number of genes that were identified for the first time, whereas gray bars indicate the number of genes that were identified at earlier time points. (c) Relative expression profiles of the O_2_-responsive genes after shifting to reoxygenation. The expression values of each time point were normalized to that of time zero. The scale bar to the left denotes 20 genes, and the color bar at the bottom indicates the degree of gene expression change relative to time zero. (d) Quantitative RT-PCR validation of the top ten O_2_-responsive genes at their respective times of maximal response.

Next, principal component analysis (PCA) was applied to examine the reproducibility between different replicates and the times when specific genes were activated. As shown in Figure 1a, replicates of the same time point aggregated together, indicating high reproducibility of our data. Also, different time points distributed sequentially according to the amount of time under reoxygenation. The time points of 8 h, 12 h, and 24 h were clustered together, indicating similar gene expression patterns at later time points.

### Dynamic responses of gene expression profiling upon reoxygenation

To quantitatively characterize the O_2_-responsive genes at each time point during acclimatization to reoxygenation, statistical analysis (Student's *t*-test) of each time point versus time 0 was applied for each gene. The number of genes that were significantly different (*P*<0.0001) from the hypoxia control were plotted in Figure 1b. The numbers of O_2_-responsive genes, including both up- and down-regulated genes, were 0 at 1 h, 17 at 4 h, 44 at 8 h, 49 at 12 h, and 35 at 24 h. At 8 h, only 7% of genes (3/44) had been identified at 4 hours, whereas, 22% of genes (11/49) at 12 h had been identified at 4 or 8 hours. Thus, this result showed that transcriptional responses were activated between 8 and 12 h after reoxygenation, and then diminished at 24 h after reoxygenation.

In order to comprehend the expression profiles of these O_2_-responsive genes, their expression values at each time point were normalized to that of time 0 (Figure 1c). The heatmap showed that, in general, the intensity of up- or down-regulated genes increased as cells stayed longer under reoxygenation. Next, the 10 genes with the largest expression changes upon reoxygenation were selected to validate the microarray results using quantitative RT-PCR. As shown in Figure 1d, the expression values of these genes, except one, at the time points with maximal response were very consistent with the microarray results.

### Pathway analysis of genes responsive to reoxygenation

In order to understand which pathways were involved in adaptation to reoxygenation, pathway analysis was performed using the NCI-Nature Pathway Interaction Database [17]. Among the 127 identified genes, pathway analysis revealed that, as expected, the most significantly (*P*<0.01) enriched pathway was the HIF-1-alpha transcription factor network, and the second most significant pathway was validated targets of C-MYC transcriptional activation (Table 1). Furthermore, to investigate which pathway was activated at each time point, pathway analyses were done separately using O_2_-responsive genes identified at each time point. The results showed that genes activated at 8 h were involved in validated targets of C-MYC transcriptional activation (Table 1). Genes activated at 12 h were mainly involved in the HIF-1-alpha transcription factor network, ceramide signaling pathway, and coregulation of androgen receptor activity.

**Table 1:** *P*-values of pathways enriched in differentially expressed genes responsive to reoxygenation.

Enriched pathway*	Genes expressed at any time point (n = 127)	Genes expressed at the following time points:				
		1 h (n = 0)	4 h (n = 17)	8 h (n = 44)	12 h (n = 49)	24 h (n = 35)
HIF-1-alpha transcription factor network	2.02E-03	- †	-	-	6.27E-03	-
Validated targets of C-MYC transcriptional activation	4.19E-03	-	-	3.07E-03	-	-
Ceramide signaling pathway	-	-	-	-	3.56E-03	-
Coregulation of androgen receptor activity	-	-	-	-	5.57E-03	-

*Pathway analysis was done using the NCI-Nature Pathway Interaction Database [17].

† *P*-value >0.01.

### Down-regulation of NDRG1 promotes cell migration under reoxygenation

**Figure 2:**
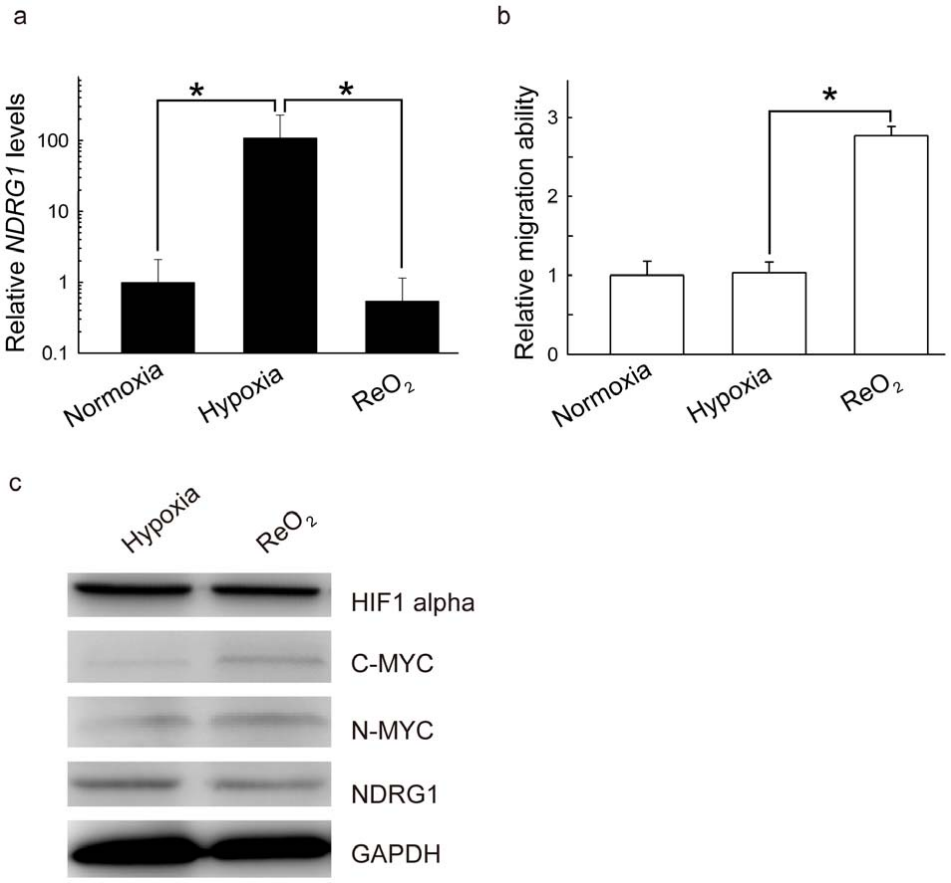
Down-regulation of *NDRG1* correlates with an increase of MCF-7 migration under reoxygenation. (a) Relative expression levels of *NDRG1* under different O_2_ conditions. The mRNA levels of *NDRG1* measured by RT-PCR were first normalized by 18s rRNA, and then compared to those in normoxia (*P<0.01). (b) Relative migration ability of MCF-7 under different O_2_ conditions. A transwell assay was used to measure MCF-7 migration. Migration ability was expressed as fold changes relative to normoxia. (c) Western blots of HIF1α, C-MYC, N-MYC, and NDRG1 in hypoxia and reoxygenation. GAPDH was the loading control.

Since *NDRG1*, which is regulated by the MYC signaling pathway, had the greatest change in expression following reoxygenation, and that these reoxygenation genes were enriched in validated targets of C-MYC transcriptional activation, we wanted to further investigate the response of *NDRG1* to reoxygenation. It was not clear whether *NDRG1* could affect the metastatic ability of tumor cells. Therefore, transwell assays were conducted to examine the migration ability of MCF-7 cells at different O_2_ concentrations. As shown in Figure 2, the transcript levels of *NDRG1* were significantly decreased upon reoxygenation (Figure 2a), whereas the migration ability of MCF-7 significantly increased (Figure 2b). A western blot confirmed that C-MYC and N-MYC increased, and NDRG1 decreased, under reoxygenation conditions (Figure 2c). These results indicate that *NDRG1* can affect migration of transformed cells via the MYC signaling pathway.

**Figure 3:**
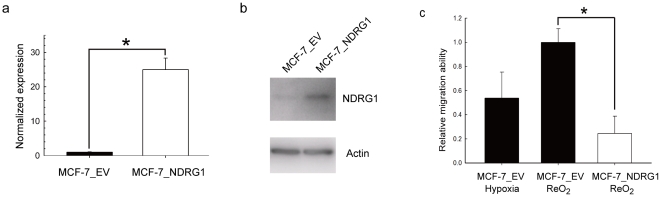
Overexpression of NDRG1 inhibits MCF-7 migration under reoxygenation. (a) Quantitative RT-PCR analysis of *NDRG1* overexpression in MCF-7. The mRNA levels of *NDRG1* were normalized by 18s rRNA. EV: empty vector. (b) Western blotting of overexpressed NDRG1. Protein from whole cell lysates was blotted with NDRG1-specific antibody, and β-actin was the loading control. (c) Relative migration ability of MCF-7 cells after overexpressing NDRG1. Migration ability was expressed as fold changes relative to MCF-7 under reoxygenation.

Next, since *NDRG1* was down-regulated upon reoxygenation, we overexpressed *NDRG1* in MCF-7 cells to investigate its physiological function. To confirm overexpression, mRNA and protein levels of NDRG1 were examined by quantitative RT-PCR (Figure 3a) and western blotting (Figure 3b). The transcript and protein levels of NDRG1 in NDGR1-transfected cells were significantly higher than those in cells transfected with the empty vector control. MCF-7 cells transfected with *NDRG1* or empty vector were then inoculated into transwells for a second round of cell migration assays under reoxygenation. The results showed that overexpression of NDRG1 significantly (*P*<0.001) inhibited cell migration under reoxygenation (Figure 3c).

### Prediction of MYC-associated transcription factors and hypoxia-related microRNAs in regulating *NDRG1*

In order to understand the mechanisms regulating *NDRG1* expression, we used bioinformatics tools and a literature survey to predict the binding motifs of MYC-associated transcription factors in the promoter of *NDRG1* and the binding sites of hypoxia-related microRNAs (miRNAs) in the 3′UTR. Using MatInspector and criteria stated in the Materials and Methods, 170 binding motifs of transcription factors were identified in the promoter of *NDRG1*. Among these transcription factors, MYC-associated transcription factors that were reported previously were selected. These transcription factors included E2F-MYC activator, MYC associated zinc fingers, and E-box binding factors. Their binding motif, location, and sequence logo are listed in Table S1. These results indicted *NDRG1* might be regulated by these transcription factors, although more experiments are warranted.

Lastly, the expression levels of miRNAs are known to change under hypoxia. To investigate the possibility of *NDRG1* being subject to miRNA regulation under different O_2_ conditions, binding sites of hypoxia-related miRNAs were searched in the 3′UTR of *NDRG1*. The search criteria allowed one mismatch, wobble, deletion, or gap between the second and seventh nucleotides of the miRNAs. As shown in Table S2, six binding sites for the seed regions of four hypoxia-related miRNAs—miR-25, miR-93, miR-106a, and miR-210—were identified in the 3′ UTR of *NDRG1*, suggesting that *NDRG1* could be regulated by these four miRNAs. This result could be used to design experiments investigating the post-transcriptional regulation of *NDRG1* by miRNAs.

## Discussion

Several studies have reported that tumor cells display increased drug resistance and metastatic potential after exposure to hypoxia/reoxygenation insults [13], [15]. Although cellular adaptations to hypoxia are well documented, little is known about adaptive mechanisms to reoxygenation. Here, we examined the dynamics of genome-wide gene expression during reoxygenation, and found that the differentially expressed genes were involved in the HIF-1-alpha transcription factor network and C-MYC transcriptional activation.

In this study, principal component analysis of the oxygen-responsive genes showed high reproducibility over time. Based on the number of O_2_-responsive genes at different time points, the active period of transcription in response to reoxygenation appears to be between 8 and 12 hours. Furthermore, pathway analysis revealed that the O_2_-responsive genes at 12 hours were involved in the HIF-1-alpha transcription factor network, the ceramide signaling pathway, and coregulation of androgen receptor activity. It is not surprising that the HIF-1-alpha transcription factor network was involved in reoxygenation, because it has been reported in a similar situation, i.e., irradiation. Following radiotherapy, tumor reoxygenation leads to nuclear accumulation of HIF-1 in response to reactive oxygen species [18]. One of genes, namely *NDRG1*, in the HIF-1-alpha transcription factor network draw our attention because it had the greatest change in expression following reoxygenation.

*NDRG1* is expressed ubiquitously in tissues stimulated under a wide variety of stresses and cell growth-regulatory conditions, such as hypoxia [19], [20], DNA damage [21], cellular differentiation [22], [23], [24], proliferation and growth arrest [23]. It has been reported that *NDRG1* is strongly up-regulated under hypoxic conditions. An oncogenic and tumor-promoting role of *NDRG1* has also been reported, because it was overexpressed in various human cancers, including lung, brain, skin, kidney, and breast cancers [25], [26]. However, *NDRG1* functioned as a metastatic suppressor in prostate and colon cancers [24], [27]. The contradictory roles of *NDRG1* in cancer remained to be clarified, although they might be explained by its multiple cellular localizations and complex regulation by diverse physiological and pathological factors. Recently, Toffoli et al. indicated that *NDRG1* can be induced under intermittent hypoxia to promote cell migration [28]. Several studies also suggested that *NDRG1* is induced by hypoxia and associated with metastasis, but the regulatory mechanism of *NDRG1* remains elusive and its function under reoxygenation is still unclear.

*NDRG1* had the maximal transcriptional response to reoxygenation in this study, which we felt warranted further investigation. We observed that the expression of *NDRG1* had an inverse relationship with cell migration upon reoxygenation. These results implicate NDRG1 as a metastasis suppressor, consistent with the findings of Maruyama et al. [8]. The discrepancy between our results and those of Toffoli et al. [28] may be due to different type of cells and experimental settings.

**Figure 4:**
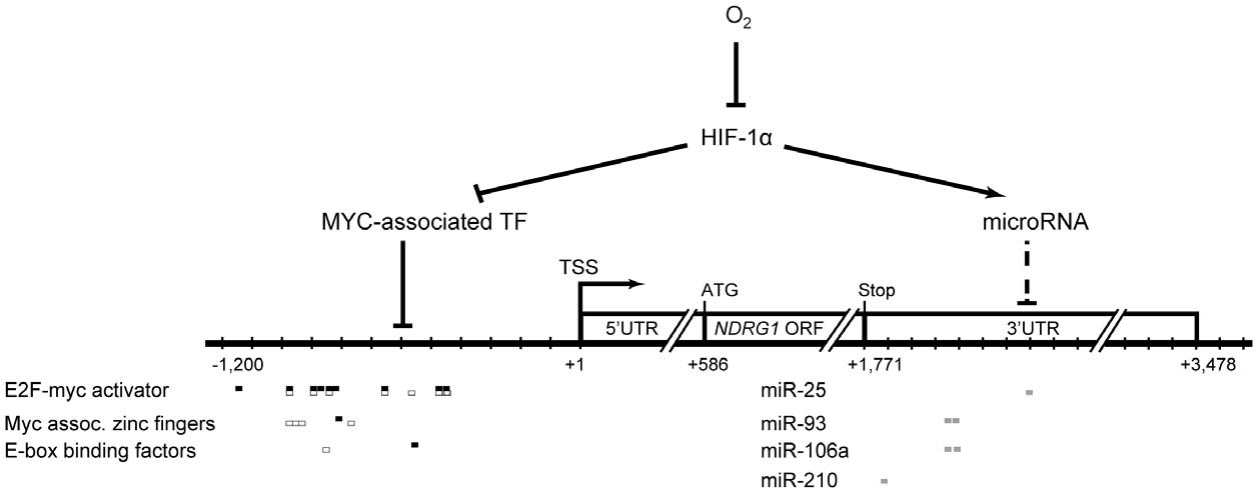
The proposed model of *NDRG1* regulation by MYC-associated transcription factors and miRNAs in response to changes in O_2_ concentration. TSS: transcription start site. White box: transcription factor binding site at + strand; black box: transcription factor binding site at - strand; gray box: miRNA binding site.

In order to understand more fully the possible regulatory mechanisms of *NDRG1* under reoxygenation, *in silico* sequence analysis was performed to predict DNA binding motifs of transcription factors in the promoter of *NDRG1*. Among the MYC-associated binding motifs identified, zinc finger proteins, E2F-MYC activator/cell cycle regulators, and E-box binding factors could affect gene expression [29], [30], [31], [32]. These candidate transcription factors can be further validated by constructing various promoters using luciferase assays. Furthermore, the expression levels of several miRNAs have been shown to change in hypoxia [33], [34], [35]. In particular, miR-210 is induced during hypoxia via a HIF1-dependent mechanism, and the expression of miR-210 had a strong correlation with the expression of *NDRG1* [34]. Therefore, we hypothesized that the expression of *NDRG1* was also regulated by miRNAs. Indeed, the binding sites of the seed regions of four hypoxia-related miRNAs (miR-106a, miR-93, miR-25, and miR-210) were identified in the 3′UTR of *NDRG1*. Therefore, we proposed a working model based on the bioinformatic prediction and literature survey (Figure 4). This model provides a framework for future biological experiments.

## Materials and Methods

### Cell culture

Human breast cancer cell line MCF-7 was obtained from Bioresource Collection and Research Center (Hsiuchu, Taiwan). MCF-7 cells were maintained in Dulbecco's modified Eagle medium (DMEM, Invitrogen Life Technologies, Carlsbad, CA) containing 1.5 g/L sodium bicarbonate supplemented with 10% (v/v) fetal bovine serum (Hyclone, Gibco) and with 1% antibiotic solution (Invitrogen Life Technologies, Carlsbad, CA). For hypoxic cultures, cells were incubated in a hypoxia chamber (InVivO_2_-200, Ruskinn Technology, Leeds, UK) for 24 h with a gas mixture containing 5% CO_2_, 95% N_2_ at 37°C. The oxygen concentration in the hypoxia chamber was maintained at 0.5%. After 24 h of hypoxic growth, cells were incubated in a well humidified incubator with 5% CO_2_ and 95% room air at 37°C. Six samples were collected respectively at 0, 1, 4, 8, 12 and 24 h after reoxygenation. The cells were washed with cold PBS, flash-frozen in liquid N_2_, and stored at −80°C for later RNA isolation. Each experiment was carried out in triplicate.

### RNA extraction

Total RNA was extracted with TRIzol Reagent (Invitrogen, Carlsbad, CA) and was purified by RNeasy Micro cleanup kit (Qiagen, Valencia, CA) according to the manufacturer's instructions. RNA concentration and quality were determined using a NanoDrop ND-1000 spectrophotometer (NanoDrop Technologies, Wilmington, DE) and an Agilent 2100 Bioanalyzer (Agilent Technologies, Palo Alto, CA), which calculates an RNA integrity number (RIN). Total RNA (500 ng) with A_260_/A_280_ = 1.7–2.1 and RIN>7.0 were used to synthesize the first strand cDNA via reverse transcription.

### Illumina human whole-genome expression beadchips

The total RNA was primed with the T7 Oligo(dT) primer and amplified by Illumina TotalPre RNA Amplification Kit (Ambion Inc., Austin, TX) to synthesize the cDNA containing a T7 promoter sequence. Following the first strand cDNA synthesis, second strand cDNA was synthesized by converting the single-stranded cDNA into a double-stranded DNA (dsDNA) template for transcription. The reaction employed DNA polymerase and RNase H to simultaneously degrade the RNA and synthesize second strand cDNA. The double-stranded cDNA then underwent a clean-up process to remove excess RNA, primers, enzymes, and salts that would inhibit in vitro transcription. Thereafter, in vitro transcription was conducted using the double-stranded cDNA as a template and T7 RNA polymerase to synthesize multiple copies of biotinylated cRNA. After amplification, the cRNA was mixed with an equal volume of hybridization buffer and hybridized to Illumina Human-6 v3 BeadChips (Illumina, San Diego, CA) at 58°C for 16 h. After hybridization, the BeadChip was washed and stained with streptavidin-Cy3 dye. The intensity of the bead's fluorescence was detected by the Illumina BeadArray Reader, and the results were analyzed using BeadStudio v3.1 software. All data is MIAME compliant and that the raw data has been deposited in a MIAME compliant database. Microarray data of this study have been submitted to the GEO (Gene Expression Omnibus) database (accession number GSE30019).

### Data mining and statistical analysis

After scanning, the intensity data of Illumina Human-6 v3 BeadChips were analyzed by the commercial software Partek® (Partek, St. Charles, MO) for mRNA expression analysis. Background-adjusted signals were normalized by a quantile normalization algorithm, which normalized the probe intensities based on the intensity distribution among all slides. After normalization, Principal Component Analysis (PCA), which reduces high dimensional data into a 2D graph, was utilized to evaluate the similarity of the gene expression profiles. In order to identify differentially expressed genes, t-tests examining the expression levels of every time point after reoxygenation versus that of time zero were utilized. Genes whose *P*-value of three replicates at one or more time points was <10^−4^ were identified and defined as O_2_-responsive genes. The Genesis program [36] was used to generate a visual representation of expression profiles. Furthermore, NCI-Nature Pathway Interaction Database [17] was applied to identify the biological functions of the differentially expressed genes.

### Overexpression of *NDRG1* in MCF-7

The human *NDRG1* gene was inserted between the *EcoR I* and *BamH I* sites of the eukaryotic expression vector pcDNA3.1+ (Invitrogen, Carlsbad, CA). MCF-7/NDRG1 cells were created by transfection of MCF-7 cells with pCDNA3.1+ encoding the *NDRG1* gene using lipofectamine 2000 (Invitrogen, Carlsbad, CA). MCF-7/NDRG1 cells were then selected by 200 µg/ml of Zeocine for two weeks. The mRNA expression of *NDRG1* was examined by quantitative real-time PCR, and NDRG1 protein expression was examined by western blotting.

### Quantitative reverse-transcription PCR

Total RNA was extracted using TRIzol Reagent (Invitrogen, Carlsbad, CA) according to manufacturer's instructions. Reverse transcription of total RNA was performed with a High Capacity cDNA RT Kit (Applied Biosystems, Foster City, CA) using random primers and 1 µg total RNA as template. The reaction mixture was incubated at 25°C for 10 min, 37°C for 2 h and 85°C for 5 sec. Real time PCR was detected by SYBR Green (Sigma) and was performed using the ABI 7300 (Applied Biosystems, Foster City, CA). The reactions were performed using the following program: 40 cycles of denaturing at 95°C for 15 sec and 1 min of annealing and elongating at 60°C. For each cDNA sample, an internal control, 18s rRNA, was also measured by the SYBR Green probe to ensure comparable amounts of cDNA in all wells. Relative expression of NDRG1 compared with 18s rRNA in each sample was calculated (△Ct) and relative expression of NDRG1 among samples was determined by calculating the difference in △Ct between samples (△△Ct). All measurements were made in triplicate (5 ng of total RNA per well).

### Western blotting

Whole cell extracts were prepared using RIPA lysis buffer supplemented with 1% Nonidet P-40 (NP-40) and Mini Protease Inhibitor Cocktail Tablets (Roche, Mannheim, Germany). Cell debris was collected by centrifugation at 8,000 x g at 4°C for 20 minutes. Protein concentration was measured by the bicinchoninic acid method (BCA assay), and 20–50 µg protein were loaded on a 10% denaturing sodium dodecyl sulfate polyacrylamide gel. After electrophoresis, protein was electrophoretically transferred to PVDF membranes overnight at 55 mA. The membranes were blocked with Tris-Buffered Saline Tween-20 (TBST) with 5% non-fat powdered milk at room temperature for an hour. Detection of specific proteins was done by probing membranes with primary antibodies in TBS with 0.1% Tween-20 for 1.5 hours at room temperature. These antibody included NDRG1 (AbCam, 1∶500), HIF1α (Millipore, 1∶1000), C-MYC (Millipore, 1∶1000), N-MYC (Millipore, 1∶250), and loading control GAPDH (GeneTex,1∶10000) or β-actin (1∶5000). After incubation with the horseradish peroxidase-conjugated IgG secondary antibodies (1∶5000), the immunoreactivity was visualized by enhanced chemiluminescence with Luminol Reagent (Bio-Rad Laboratories, Richmond, CA, USA).

### Cell migration assay

Migration assays were carried out using 24-well transwell migration chambers (Corning, Corning, New York, USA) with 8 µm pore size polyethylene membranes. Cells were first starved 24 h and were harvested by Accutase (PAA Laboratories, Linz, Austria). The upper chambers were inoculated with 5×10^4^ cells/well in 0.1 ml serum-free DMEM cell solution, and lower chambers were filled with 0.6 ml DMEM containing 10% FBS as chemoattractant. Cells were allowed to migrate for 24 h at 37°C. For measuring the migrated cells, 2 µg/ml Calcein-AM (Trevigen, Gaithersburg, MD, USA)/Cell Dissociation Solution (Trevigen) was added into the lower chamber. After incubation at 37°C for 60 min, inserts were removed and plates were read at 485 nm for excitation and 520 nm for emission. Cell numbers were calculated by comparing the absorbance to the standard curve. MCF-7 cells transfected with empty vector were used as the control for each experiment.

### *In silico* analysis of *NDRG1*

To identify potential binding motifs of MYC-associated transcription factors in the promoter of *NDRG1*, the MatInspector program was utilized [37]. Two predefined groups of transcription factors, including general core promoter elements and vertebrates, were analyzed in the matrix library version 8.3 using the following parameters: (1) matrix families were matched instead of individual sequences; (2) core similarity was at least 0.75; (3) matrix similarity was optimized. The core region was defined as four consecutive nucleotides with the highest conservation scores [38]. The core and matrix similarity were calculated by comparing the query sequence with the most conserved nucleotide in the matrix. After identifying the potential transcription factor candidates, the MYC-associated transcription factors were further selected based on a literature survey.

For identifying the binding sites of hypoxia-related miRNAs, the *NDRG1* 3′UTR sequence was downloaded from UCSC's Genome browser (http://genome.ucsc.edu/cgi-bin/hgGateway; GRCh37/hg19 assembly), and was aligned to hypoxia-related miRNAs. The criteria for searching binding sites of the seed region were allowing one mismatch, wobble, deletion, or gap between the second and seventh nucleotides of hypoxia-related miRNAs.

## Supporting Information

Table S1 **Predicted binding sites of MYC associated transcription factors in the promoter of** ***NDRG1*** **.**
(DOC)

Table S2 **Predicted binding sites of miRNAs in the 3′UTR of** ***NDRG1*** **.**
(DOC)
