# Supplementary material for: Down-Regulation of NDRG1 Promotes Migration of Cancer Cells during Reoxygenation
Source: PLoS One. 2011 Aug 30;6(8):e24375. doi: 10.1371/journal.pone.0024375 (PMC3166165; doi:10.1371/journal.pone.0024375)
Supplement: Table S1 — Predicted binding sites of MYC associated transcription factors in the promoter of NDRG1 . (DOC) [file pone.0024375.s001.doc]

Table S1. Predicted binding sites of MYC associated transcription factors in the promoter of *NDRG1*

| **Name**  **(Sequence logo)** | | **Binding Sequence** |  | **Strand** |  | **Start position*** |  | **End position*** |
| --- | --- | --- | --- | --- | --- | --- | --- | --- |
|  | **E2F-MYC activator**  **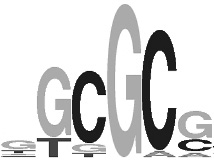** | agaggGCGCgggcttac | - | | -1152 | | -1168 | |
|  |  | tatagGCGCcgcggccc | - | | -990 | | -1006 | |
|  |  | cccggGCGCgccgggct | - | | -888 | | -904 | |
|  |  | cggcaGCGCgcggcggg | - | | -860 | | -876 | |
|  |  | gcctgtTGCGcaagcag | - | | -826 | | -842 | |
|  |  | tagggGCGCggccggga | - | | -806 | | -822 | |
|  |  | catgtGCGCgctcgtgc | - | | -669 | | -685 | |
|  |  | cgctttCGCCcgacagg | - | | -478 | | -494 | |
|  |  | ccgtgtcgcCAAAgctc | - | | -440 | | -456 | |
|  |  | ccgcgGCGCctataaag | + | | -993 | | -1009 | |
|  |  | gcccgGCGCgcccggga | + | | -889 | | -905 | |
|  |  | ccgccGCGCgctgccgg | + | | -861 | | -877 | |
|  |  | tgcttgCGCAacaggcg | + | | -827 | | -843 | |
|  |  | cacgaGCGCgcacatga | + | | -670 | | -686 | |
|  |  | gcgtgGCGCaacgagac | + | | -578 | | -594 | |
|  |  | ctgtcgGGCGaaagcga | + | | -479 | | -495 | |
|  |  | ctttgGCGAcacggaag | + | | -443 | | -459 | |
|  |  | agctttGGCGacacgga | + | | -441 | | -457 | |
|  | **MYC associated zinc fingers**  **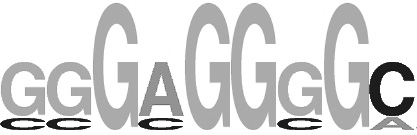** | ccgggaGGGGcat | - | | -799 | | -811 | |
|  |  | gggggcGGGGccg | + | | -983 | | -995 | |
|  |  | gggcGAGGcgcgg | + | | -972 | | -984 | |
|  |  | ctggGAGGggcga | + | | -965 | | -977 | |
|  |  | ctcgGAGGggact | + | | -758 | | -770 | |
|  | **E-box binding factors** | gcgCCACgccctg | - | | -574 | | -586 | |
|  |  | ccgccgCGCGctg | + | | -861 | | -873 | |

* Position relative to the transcription start site.
