# Supplementary material for: Down-Regulation of NDRG1 Promotes Migration of Cancer Cells during Reoxygenation
Source: PLoS One. 2011 Aug 30;6(8):e24375. doi: 10.1371/journal.pone.0024375 (PMC3166165; doi:10.1371/journal.pone.0024375)
Supplement: Table S2 — Predicted binding sites of miRNAs in the 3′UTR of NDRG1 . (DOC) [file pone.0024375.s002.doc]

Table S2. Predicted binding sites of miRNAs in the 3’UTR of *NDRG1*

| **Name** | **Alignment**^*^ | **Start position** ^†^ | **End position*** |
| --- | --- | --- | --- |
| **hsa-miR-25** | 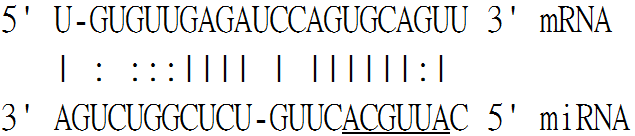 | 2,462 | 2,483 |
| **hsa-miR-93** | 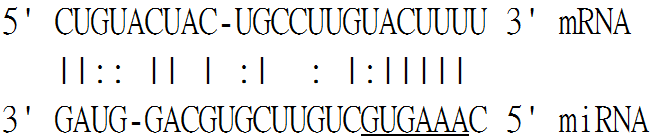 | 2,103 | 2,125 |
|  | 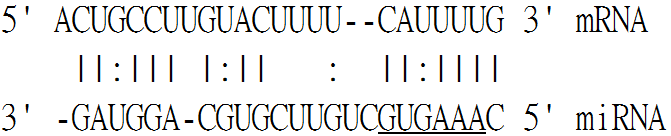 | 2,096 | 2,118 |
| **hsa-miR-106a** | 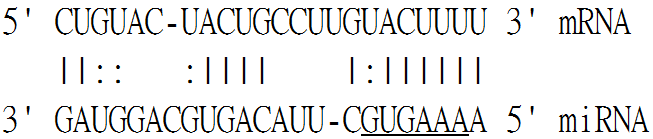 | 2,103 | 2,125 |
|  | 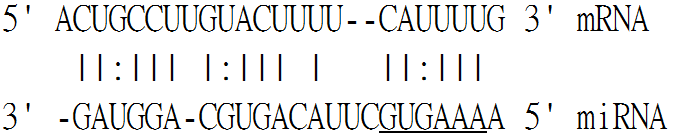 | 2,096 | 2,118 |
| **hsa-miR-210** | 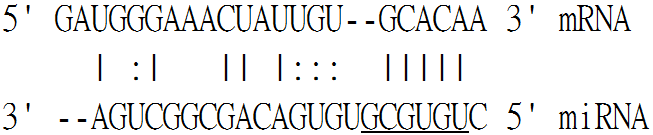 | 1,845 | 1,866 |

^*^ The seed region of each miRNA is marked by an underline.

^†^ Position relative to the transcription start site.
